# Supplementary material for: A simple model for the evolution of temperature-dependent sex determination explains the temperature sensitivity of embryonic mortality in imperiled reptiles
Source: Conserv Physiol. 2021 May 10;9(1):coab020. doi: 10.1093/conphys/coab020 (PMC8111383; doi:10.1093/conphys/coab020)
Supplement: Supplementary_table_coab020 [file supplementary_table_coab020.docx]

**Table S1:** Summary of Model B, the top-ranked model fitting embryonic mortality as a function of *∆T_TPiv_*, using all available data (n = 199)

| Parameter | Effect Type | Estimate^a^ | SE |
| --- | --- | --- | --- |
| StudyID | Random | 1.24 | - |
| Residual | Random | 24.9 | - |
| Intercept | Fixed | 1.96 | 0.445 |
| bs(Dev.Tpiv) | Fixed | -5.73 | 1.07 |
| bs(Dev.Tpiv)^2^ | Fixed | -3.54 | 0.784 |
| bs(Dev.Tpiv)^3^ | Fixed | 0.687 | 0.920 |

^a^ Estimates for random effects are variance estimates

^b^ Standard error of the estimate
